# Austerity and health: the impact in the UK and Europe

**DOI:** 10.1093/eurpub/ckx167

**Published:** 2017-10-10

**Authors:** David Stuckler, Aaron Reeves, Rachel Loopstra, Marina Karanikolos, Martin McKee

**Affiliations:** 1 Dondena Research Centre, University of Bocconi, Milan Italy; 2 Department of Sociology, University of Oxford, Oxford, UK; 3 Department of Social Inequality, London School of Economics, London, UK; 4 Department of Public Health and Policy, London School of Hygiene & Tropical Medicine, London, UK

## Abstract

Austerity measures—reducing social spending and increasing taxation—hurts deprived groups the most. Less is known about the impact on health. In this short review, we evaluate the evidence of austerity’s impact on health, through two main mechanisms: a ‘social risk effect’ of increasing unemployment, poverty, homelessness and other socio-economic risk factors (indirect), and a ‘healthcare effect’ through cuts to healthcare services, as well as reductions in health coverage and restricting access to care (direct). We distinguish those impacts of economic crises from those of austerity as a response to it. Where possible, data from across Europe will be drawn upon, as well as more extensive analysis of the UK’s austerity measures performed by the authors of this review.

## Introduction

Austerity is a massive experiment on people of Europe. It was imposed in the aftermath of the Great Recession of 2007, precipitated by the collapse of the housing bubble in the USA. In 2009, gross domestic product (GDP) fell in real terms in all countries of the European Union (EU) except Poland; the mean decrease was 4.3%, but losses ranged from 1.9% in Cyprus to 17.7% in Latvia.^1^ Between 2007 and 2010, unemployment increased substantially and rapidly, e.g. by 3% in Portugal, Slovakia and Bulgaria; 4% in Denmark, Hungary and Greece; 5% in Iceland; 9% in Ireland; 12% in Spain and Estonia; 13% in Latvia and 14% in Lithuania. By 2016, economic output had only just returned to pre-crisis levels.

How best to promote economic recoveries is a topic of ongoing debate. During the initial onset of financial crisis, politicians in nations with significant financial sectors, particularly the USA and UK, along with Sweden and Germany tended to implement large stimulus packages. These were used to bail out banks, absorbing their debts into the public sector’s balance sheet. In parallel, however, the economic slowdown was leading to job losses and falling incomes, in turn causing drops in consumer spending and associated tax revenues. These forces, when combined with large bailout packages for the financial sector, generated large rises in government deficits (where annual government spending exceeds revenues) and, resultantly, increasing national public debts.

Two broad options exist to achieve debt reduction: invest to promote economic growth and thus boost government revenues for debt repayment, or reduce government spending to free up revenue for debt repayment. The European Commission, European Central Bank and International Monetary Fund (the so-called ‘troika’), along with leaders of many European nations, placed an explicit priority on the latter approach to deficit reduction. In theory, deficit reduction can be achieved by either raising taxes or reducing expenditure. When combined these activities are sometimes referred to as ‘fiscal consolidation’. In practice, the majority of deficit reduction policies (>80%) in Europe involved budget cuts rather than tax increases.^2^ Consequently, for coherence we refer to these policies as ‘austerity’.

This review aims to assess: what impacts have these austerity measures had on health and well-being, and what has helped to buffer them?

Austerity is now known to be clearly regressive (Box 1). While there is now an extensive literature on the economics of austerity, much less is known about their impact on health and well-being. At the time of this writing, in 2017, more than half a decade has passed since the initial experiments with austerity. A review in the *The Lancet* noted that ‘public health voices have been largely absent from the debate about how to respond’.^1^ It also pointed out that, in the EU, the Directorate-General for Health and Consumer Protection of the European Commission, despite its legal obligation to assess the health effects of EU policies, has not assessed the effects of the troika’s drive for austerity, and has instead limited EU commentary to advice about ‘how health ministries can cut their budgets’.

Conceptually, austerity can impact on health through two mechanisms: (i) a ‘social risk effect’ of increasing unemployment, poverty, homelessness and other socio-economic risk factors, while cutting effective social protection programmes that mitigate their risks to health (with the latter being an interaction between austerity and economic shocks); and (ii) a ‘healthcare effect’ through cuts to healthcare services, as well as reductions in health coverage and restricting access to care.

The rest of this paper (adapted from a forthcoming book chapter) performs a brief review of evidence about these two channels, starting with the indirect effects, on unemployment, homelessness and food security. Importantly, we seek to distinguish those impacts of economic crises from those of austerity as a response to it. Where possible, data from across Europe will be drawn upon, as well as more extensive analysis of the UK’s austerity measures performed by the authors of this review.

## Effects on social risk and protective factors

### Unemployment

Austerity measures have sought to make savings by reducing public sector employment; resulting job losses can be expected to increase depression and suicide rates. Taking the UK as an example, according to the Office of National Statistics, there were over 500 000 public sector job losses between June 2010 and September 2012, of which over 35% were in the North of England.^2^ The regional pattern of job losses correlates with changes in suicides; a 20% rise was observed in those regions most affected by austerity: the North-East, the North-West, and Yorkshire and the Humber, but a decline in London, where unemployment fell.

Austerity has, in many nations, been achieved by reducing social spending on the unemployed. One means is to tighten eligibility for unemployment insurance. The UK has done this through expanding its punitive policies of ‘sanctions’—cutting benefits when an unemployment support recipient fails to meet strict conditions, including evidence of actively seeking work. Qualitative research has found that these policies increase risks of hunger and depression, and quantitative studies identify that they increase risks of food insecurity and homelessness.

### Homelessness

Homelessness increases risks of infectious disease, physical harm, food insecurity, multiple morbidities and premature mortality. The application of austerity to housing support and subsidies, at a time of rising housing costs throughout much of Europe, has contributed to a growing burden of homelessness and less severe forms of housing insecurity. The European Federation of National Organisations Working with Homeless People (FEANTSA) found that 60% of homelessness organizations experienced cuts in 2011. FEANTSA further argued that ‘need to introduce austerity measures has been used as an excuse by governments not to commit to ambitious homelessness strategies’, citing how Poland abandoned its draft strategy during the crisis.^3^

Budget reductions can trigger increasing risks of homelessness. FEANTSA’s analysis across 30 EU countries found that austerity increased vulnerability of families with children to evictions and repossessions.^3^ One cross local area analysis of 323 authorities in the UK found that budget reductions in housing services and emergency housing assistance payments were strongly correlated with rising rates of people seeking emergency aid for housing.^4^

### Food insecurity

Images of people queuing for food aid recall scenes from the Great Depression, but have now come to characterise many European nations subjected to austerity. In 2016, the UK charity, the Trussell Trust, provided emergency food assistance to over 1 million adults and children, a marked rise from prior to the period of austerity in 2010.^5^ Greek, Spanish and French charities also report marked rises in people seeking emergency food support coinciding with the introduction of austerity measures.

There is a dearth of comparative data on household food insecurity. The EU Survey on Income and Living Conditions (EU-SILC) surveys if households are able to afford meat (or a vegetarian equivalent) every second day.^6^ Across Europe, from 2005 to 2010 the proportion of people reporting an inability to afford to eat meat or equivalent declined by approximately half a percentage point each year.^7^ After 2010, when austerity measures were imposed, this trend reversed, rising from 8.7% in 2009 to 10.9% in 2012, remaining elevated thereafter (approximately an additional 13.5 million people experiencing food insecurity). While unemployment and stagnating wages have been some of the major drivers of rising food insecurity in Europe, cuts to social protection spending appear to have exacerbated the impact of these economic shocks on access to healthy diets.

### Mental health

There is now a large body of evidence on how economic hardship can beget worse mental health. One multi-country study using longitudinal data from health and retirement surveys in the USA and 13 EU countries found that job loss among 50–64 year olds, particularly when due to firm closure, was associated with a 28% increase in a depressive symptoms in the USA and of 8% in Europe.^8^ In Greece, 1-month prevalence of major depressive episode increased from 3.3% in 2008 to 8.2% in 2011 and 12.3 in 2013.^9^ Similar patterns were observed in Australia, England, Spain and the USA. In Ireland patients admitted with an episode of depression attributable to adverse economic circumstances linked to recession had higher suicide risks (but otherwise more favourable mental health outcomes) than patients with depression caused by other factors.^10^

Suicide rates often rise during periods of economic downturn. Prior to the onset of recession in 2007, suicide rates had been falling in Europe. Subsequently, this downward trend reversed, rising by 6.5% by 2009 and remaining elevated through 2011. This increase corresponds to an additional 7,950 suicides above what would be expected on past trends between 2007 and 2010.

Typically suicide rates rebound after GDP recovers. However, in many European nations, suicide rates remain elevated even where economic recovery appears to have occurred. The reasons are multiple; importantly, several socio-economic risk factors for suicide remain elevated. These include unemployment, unaffordable housing and indebtedness. One cross-national analysis investigated the role of these three risk factors across 20 EU countries, examining the association of suicide rates with rates of unemployment, unaffordable housing and indebtedness at the national level.^11^ They found that suicides were most closely associated with unemployment rates, particularly among the working-age population. However, there is now emerging evidence that these types of ‘economic suicides’ may be preventable (Box 2).

In summary, the available evidence indicates that austerity has exacerbated and prolonged the mental health risks associated with economic downturns.

### Pensioners and old-age mortality

One concerning trend is the rise in old-age mortality observed over the last few years in some European countries. In 2015, Italy witnessed the highest mortality rate since WWII; primarily due to a marked rise among those ages 75–95 years. In the same year, the UK experienced the largest annual rise in the mortality rate for 50 years. The number of deaths in the UK has been rising since 2011 (although with a transient recovery in 2014) after a steady decline from the late-1970s onwards. Like Italy, this rise has been particularly large among the elderly.

Austerity measures, rather than economic hardship per se, appear to have played a role in this rising death rate. Analysis from the UK—which examines changing patterns across local areas—finds that cuts to social care and financial support to elderly pensioners are associated with a rise in mortality among those ages 85 years and over.^12^

## Impact on health systems

### Financing and efficiency

An European-wide review identified a wide range of responses to economic downturn adopted by countries in the region.^13^ In response to fiscal pressures, many political leaders responded by reducing public health funding. The largest cuts were seen in Greece, Ireland, Latvia and Portugal. Some countries, however, adopted measures to protect their health systems, at least temporarily, or reduce the extent or impact of budget reductions. These mechanisms and factors which helped to make health systems more resilient include^13^:

- Policies to boost counter-cyclical public spending on health and other forms of social protection.
- Initial adequate levels of public spending on health.
- Maintenance of comprehensive health coverage with no gaps.
- Absent or relatively low levels of out-of-pocket payments.
- Making greater use of comparative information about the cost-effectiveness of different services and interventions, with disinvestment or selective investment where deemed appropriate.
- Political will to tackle inefficiencies and to mobilize revenue for the health sector.

The areas most affected by cuts were hospital sectors, administrative costs and prices of pharmaceuticals, as well as staff numbers and wages.

### Coverage and access to care

Key areas of health coverage, such as who is covered, for which services, and to what extent, have seen changes in most European countries during the crisis. These largely focused on increasing user fees, thus placing higher financial burden on patients. A survey of experts has indicated that policy makers often view user fees as a way to raise funding for health system budget,^13^ contrary to now extensive evidence that they yield little additional revenue, much of which is accounted for by administration costs, while impacting adversely on access to care. Cost-shifting in response to recessions has begun to erode financial protection. Compared with the situation prior to the crisis, the Survey of Health, Ageing and Retirement in Europe (SHARE) that follows up cohorts of people 50+ reveals out-of-pocket expenditures grew as well as did the proportion of people incurring catastrophic health expenditures.

Austerity appears to have had a greater impact on access than did economic crisis. Across Europe there has been an overall reversal in prior downward trends in ‘self-reported unmet medical need’, whereby people believed that they needed healthcare services but could not access them. These unmet needs had declined by 2% points in Europe between 2005 and 2009.^14^ In 2010, corresponding to when austerity measures largely began to take effect, unmet needs began to rise, increasing by 0.4% points to 3.4% in 2012 (corresponding to an additional 1.5 million Europeans). This rise in unmet medical need has been particularly pronounced in countries where the share of out-of-pocket payments is high, such as Greece, Latvia and Portugal. The latest EU-SILC survey data from these countries show that unmet need increased by 4–6% points when cuts peaked.

Austerity in healthcare can widen existing socio-economic gaps in access to services. Unmet need has progressively increased in Greece between 2008 and 2013 while the inequalities gap has widened dramatically: among the poorest income quintile, unmet need has doubled, from 7 to 14%, while among the richest income quintile it remained below 1%, with the exception of increase in 2011–12.^15^^,^^16^ Inequalities in unmet need can be further widened by job loss: a study from the USA found that losing work during the recession increased the probability of unmet need by 4% in richer families, and by more than 6% in poor ones.^17^

## Conclusions

The financial crises that began in 2007 confronted many countries with a choice. These nations could either invest to promote economic growth or to consolidate the economy with cuts to spending and tax rises. Each made different choices, with some investing in some areas while cutting others. However, some countries, those subject to conditions imposed by the troika, had no choice and were forced to implement austerity policies.

Although beyond the scope of this review, there is now a growing consensus that austerity slowed, or in some cases, prevented economic recovery. However, austerity also had important consequences for health and health services. It impacted most on those already vulnerable, such as those with precarious employment or housing, or with existing health problems. It was associated with worsening mental health and, as a consequence, increasing suicides. Yet, this was not inevitable. Those fortunate to live in countries with strong social protection systems, such as Iceland and Germany, escaped the worst of the crisis, compared with those with relatively weaker systems, such as Greece.

Looking ahead, the crisis and resulting austerity have accelerated a move to a new model of the economy, one in which power has shifted away from ordinary people and towards those with the greatest control over resources, a group who have emerged from the crisis relatively unscathed, with wealth more concentrated than ever among those at the very top of the distribution. Those without power face a future that is more precarious than ever, with a new term, zero-hour contract, entering the vocabulary in several countries and with the erosion of previous social safety nets.

There are many lessons to be learned from the experience of recent years. Some relate to economic policy, as what had become orthodoxy since the 1980s tested to destruction, forcing a relearning of lessons from the 1930s. Others relate to health and welfare policy, with the natural experiments that have taken place providing new insights into the importance of a strong welfare state. Unfortunately, it is not clear that these lessons will be learnt.

There are also lessons for researchers. At the onset of the crisis, health researchers were working in the dark. While financial data became available within weeks, or in some cases seconds, it took several years to obtain data on health. In these circumstances it was hardly surprising that the balance sheets of the banks (and the incomes of their executives) would be prioritized over the lives of the poor and marginalized. This cannot be allowed to happen again and, while this has stimulated interest in alternative, more timely sources of data, such as trends in internet searches for ‘suicide’,^18^ the research community must advocate for strengthened systems of data collection. It is ironic that Greece’s participation in the important SHARE project was terminated on cost grounds just as the crisis was hitting. However, once data did become available, the health research community rose to the challenge, drawing on a wide range of disciplines and expanding the use of innovative studies of natural experiments.

Research on financial crises and austerity has helped to define a new research agenda, now termed the political economy of health that emphasises the importance of studying the distribution of power, whether visible, hidden, or invisible, in society, and the means by which it impacts on population health. While the findings from this research will never be able to prevent another crisis, such as that experienced since 2007, they can at least help to ensure that the health consequences form part of the policy debate.

Box 1 Evidence on the socio-economic impact of austerity

There is now clear evidence that austerity is regressive, impacting most on the poor, thus widening socio-economic inequalities.^19–22^ The full scale of regressivity, however, depends on which areas of the budget are cut. In general they tend to impact more greatly on more vulnerable groups and on deprived regions within countries. An analysis by the IMF found that, historically, austerity measures that are pursued through spending-based consolidations are much worse than those based on tax- based consolidation.^21^
Progressive taxation and targeted social benefits could offset these adverse distributional effects. However, changes to the tax and benefit system in most countries have not kept pace with the cost of living (except for in Germany and Romania), leaving many people worse off. Who has been hardest hit varies greatly. In real terms, cuts have fallen hardest on wealthier groups in Portugal and Greece but in some countries the poor have taken the hardest hit (e.g. in Germany, Lithuania and to some extent Ireland).^23^ However, for vulnerable groups on the margins across all countries studied, these cuts, led to greater difficulty affording life’s necessities.

Box 2 Prevention of economic suicides

There is emerging evidence that economic suicides may be preventable. Some European countries seem to have avoided this association. For example, in Austria the suicide rate has not increased despite rising unemployment during the recession. In those nations with greater degrees of investment in active labour market programmes and unemployment insurance, the impact of job loss on suicide appears to be attenuated.^11^^,^^24^ Another protective factor identified relates to people’s ability to turn to family and friends for support. This is sometimes operationalized as a concept known as ‘social capital’, and measured as the degree to which people trust each other in a society. One Canadian study found that high social capital moderated the impact of the crisis on mental health: while financial strain led to deterioration in mental health overall, in communities with high compared with low social capital the effect was milder by a factor of around two for stress and depression.^25^

## Funding

DS is funded by a Wellcome Trust Investigator Award.

*Conflicts of interest*: None declared.
